# Causal relationships between 25-hydroxyvitamin D levels and laryngeal cancer risk: A two-sample Mendelian randomization study

**DOI:** 10.29219/fnr.v69.11489

**Published:** 2025-11-17

**Authors:** Dapeng Wang, Li Zhang, Yanfen Cui, Ruyuan Guo, Fuli Zhang, Junjie Zhang, Xiaotang Yang

**Affiliations:** 1 Department of Radiation Oncology, Shanxi Medical University/Shanxi Province Cancer Hospital/Shanxi Hospital Affiliated to Cancer Hospital, Chinese Academy of Medical Sciences/Cancer Hospital Affiliated to Shanxi Medical University, Taiyuan, P.R. China; 2 Department of Head and Neck Surgery, Shanxi Medical University/Shanxi Province Cancer Hospital/Shanxi Hospital Affiliated to Cancer Hospital, Chinese Academy of Medical Sciences/Cancer Hospital Affiliated to Shanxi Medical University, Taiyuan, P.R. China; 3 Department of Radiology, Shanxi Medical University/Shanxi Province Cancer Hospital/Shanxi Hospital Affiliated to Cancer Hospital, Chinese Academy of Medical Sciences/Cancer Hospital Affiliated to Shanxi Medical University, Taiyuan, P.R. China

**Keywords:** laryngeal cancer, 25-hydroxyvitamin D, Mendelian randomization, single-nucleotide polymorphisms

## Abstract

**Background:**

The relationship between 25-hydroxyvitamin D (25(OH)D) levels and the risk of laryngeal cancer (LC) is unclear. This study aimed to explore the causal association between 25(OH)D levels and LC risk using the two-sample Mendelian randomization (MR) analysis.

**Methods:**

Single-nucleotide polymorphisms for 25(OH)D levels and LC were extracted from published genome-wide association studies (GWAS) or the Medical Research Council Integrative Epidemiology Unit (MRC-IEU) Open GWAS project. Univariable MR and multivariable MR analyses were performed. Five MR methods including MR-Egger, weighted-median, inverse-variance weighted (IVW), simple mode, and weighted mode were applied.

**Results:**

Univariable MR analysis identified the fact that genetically predicted higher levels of 25(OH)D were associated with lower odds of LC [IVW: (odds ratio (OR) = 0.9993, 95% confidence interval (CI), 0.9987–0.9999; *P* = 0.019)]. Multivariable MR analyses suggested that genetically predicted higher levels of 25(OH)D were correlated with lower risk of LC after adjusting for pack-years of cigarette smoking (CS) [IVW: (OR = 0.9993, 95% CI, 0.9987–0.9998; *P* = 0.006)] or both pack-years of CS and weekly alcoholic drinks [IVW: (OR = 0.9993, 95% CI, 0.9988–0.9998; *P* = 0.011)], but this was not significant after adjusting only for weekly alcoholic drinks [IVW: (OR = 0.9995, 95% CI, 0.9989–1.0001; *P* = 0.077)].

**Conclusions:**

This MR analyses supported a slight protective effect of higher levels of 25(OH)D on the risk of LC.

## Popular scientific summary

- We know vitamin D is vital for bones, but can it also help prevent cancer? Our study investigated its link to laryngeal (throat) cancer using “Mendelian randomization”—a genetic approach that minimizes confounding from lifestyle factors like smoking and drinking.
- We found that individuals genetically predisposed to higher vitamin D levels had a slightly reduced risk of laryngeal cancer. Importantly, this protective association persisted even after accounting for smoking and alcohol use.
- Although the effect for any single person is modest, vitamin D deficiency is widespread globally. Since supplementation is generally safe and low-cost, maintaining adequate vitamin D levels could be a simple, low-risk public health strategy to help reduce cancer risk at the population level. Further research is needed to confirm these findings.

Laryngeal cancer (LC) is one of the most common respiratory tract tumors (1). A global cancer statistic in 2020 showed 184,615 new cases of LC and 99,840 deaths from LC (2). Five-year relative survival rates for LC patients are about 60.7%, which may be related to the high percentage of patients diagnosed at advanced stages (3). The main risk factors for LC include tobacco smoking, alcohol drinking, poor dietary habits, and human papillomavirus (HPV) infection (1, 4). Identifying controllable factors affecting the occurrence of LC remains important for LC.

Diet is a readily modifiable factor whose impact on LC has been significant (4, 5). Red meat and processed meat have been reported to increase the risk of LC (5, 6), while diets containing fruits and non-starchy greens reduce the hazard (7, 8). A number of studies have linked vitamin D (VD) or its metabolite 25-hydroxyvitamin D (25(OH)D) to cancer risk (9, 10). Pu’s study showed that increased intake of VD or high levels of circulating 25(OH)D were linked to a lower risk of head and neck cancer (9). Mai et al. found an elevated risk of early diagnosis of nasopharyngeal cancer in people with genetically predicted low 25(OH)D levels (10). However, a randomized controlled trial (RCT) found that VD supplementation failed to lead to a reduced frequency of invasive cancer than placebo (11). Two Mendelian randomization (MR) studies revealed no association of genetically predicted 25(OH)D levels with OC and pharyngeal cancer (PC) risk (12, 13). Due to the heterogeneity among different cancers, the factors affecting different cancers vary. The link between genetically predicted 25(OH)D levels and LC risk is unknown.

MR analysis uses the inherent properties of genetic variations to determine causal associations between risk factors and outcomes (14). We intended to use univariable and multivariable MR analyses to explore the casual association of the levels of genetically predicted 25(OH)D with LC risk.

## Methods

### Data source and study design

**Fig. 1:**
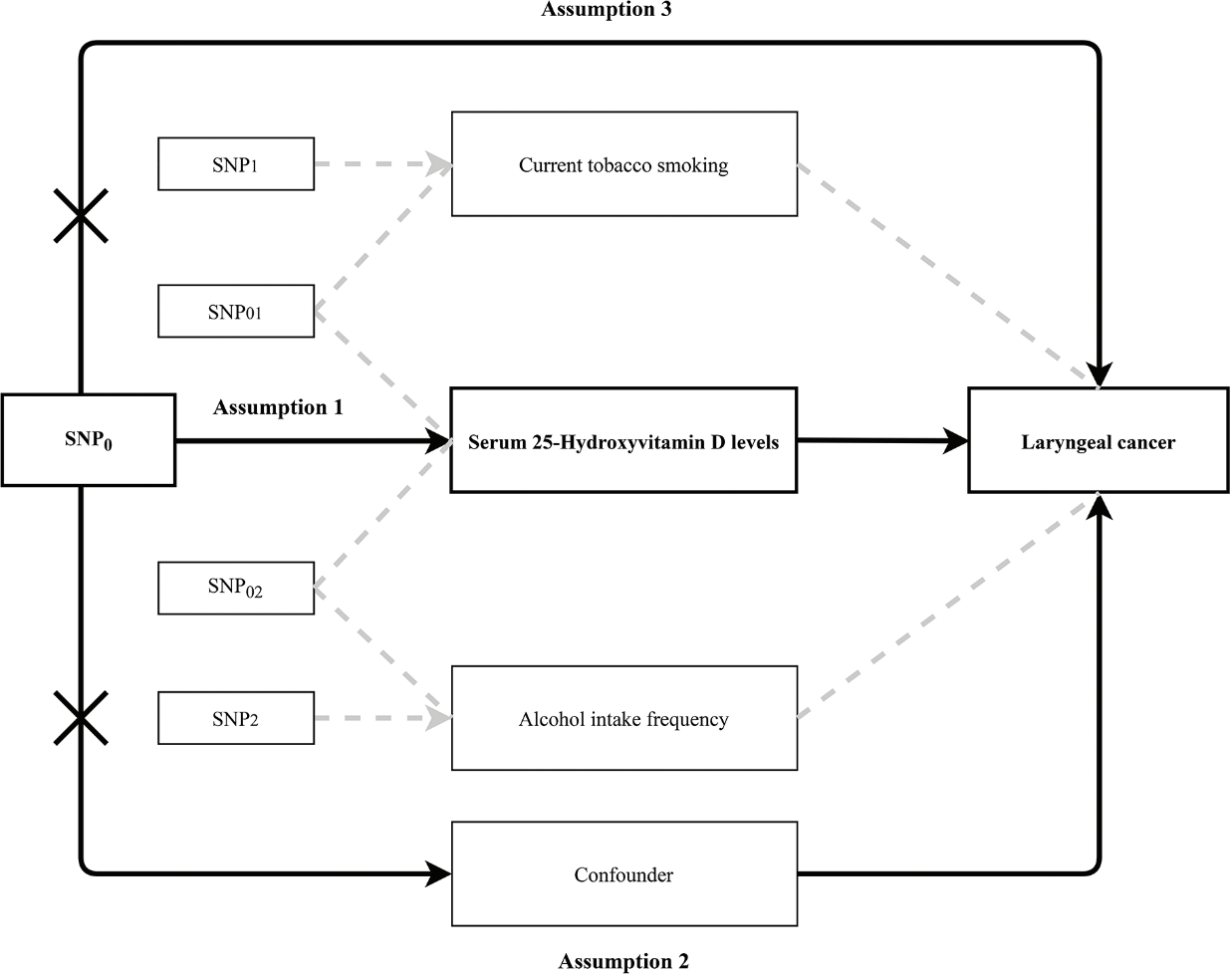
Overview of assumptions for Mendelian randomization (MR) studies. SNP0, SNPs associated with 25-hydroxyvitamin D levels only; the other SNPs, associated with 25-hydroxyvitamin D levels as well as smoking or drinking. SNP: single-nucleotide polymorphisms.

A two-sample MR analysis was conducted. Single-nucleotide polymorphism (SNPs) related to exposure and outcomes were derived from published genome-wide association studies (GWASs) or traits reported in the MRC-IEU Open GWAS project (https://gwas.mrcieu.ac.uk/datasets/) (15). MR is based on three key assumptions, specifically that (1) the SNPs were linked to the exposure; (2) SNPs in the exposure-outcome association were not linked to confounders; and (3) the SNPs only impact the outcome by exposure (Fig. 1). The present study was a secondary analysis based on data from previously published original studies, and the ethical approval and informed consent were obtained in the previously published original studies. The ethical approval and informed consent in this paper are not applicable.

### Selection of genetic instrumental variables

The GWAS summary statistics for 25(OH)D levels were obtained from a UK Biobank-based GWAS (16), which included 496,946 Europeans and identified 143 independent loci related to 25(OH)D. The GWAS summary statistics for pack years of cigarette smoking (CS) were from the MRC-IEU database, with 142,387 Europeans for smoking. The GWAS summary statistics for weekly alcoholic drinks were derived from the GWAS and Sequencing Consortium of Alcohol and Nicotine use, with 335,394 Europeans for drinking (17). The GWAS summary statistics for LC were obtained from the MRC-IEU database, 372,289 Europeans were included, of which 273 patients had LC. Detailed information on SNPs associated with exposure and outcomes is shown in Table 1.

**Table 1:** Overview of the source of exposure and outcome

Variables	GWAS ID	Year	Sample size	Number of SNPs	Population	PMID/ Consortium
Serum 25-hydroxyvitamin D levels	ebi-a-GCST90000618	2020	496,946	496,946	European	32242144
Pack years of smoking	ukb-b-10831	2018	142,387	9,851,867	European	MRC-IEU
Alcoholic drinks per week	ieu-b-73	2019	335,394	335,394	European	30643251
Laryngeal cancer	ieu-b-4913	2021	372,289	7,239,512	European	UK Biobank

GWAS: genome-wide association studies; SNP: single-nucleotide polymorphism; PMID: PubMed Unique Identifier.

The screening criteria for instrumental variables were as follows: (1) SNPs are related to exposure at the genome-wide statistical significance threshold (*P* < 5 × 10^−8^); (2) SNPs without linkage disequilibrium (LD) (*R*^2^ < 0.01, 10,000 kb clumping distance); (3) SNPs with minor allele frequency (MAF) more than 0.01. Furthermore, the *F*-statistics and variance explained (*R*^2^) were calculated to detect weak instrument bias, where *F*-statistics <10 implies that there is a weak instrument variable (18).

### Statistical analysis

Univariable MR and multivariable MR analyses were performed to analyze the relationship of genetically predicted 25(OH)D levels to LC risk, respectively. For univariable MR analysis, horizontal pleiotropy was assessed by MR-Egger regression test, with intercept *P*-value > 0.05 representing the absence of horizontal pleiotropic effects (19). MR-Egger, weighted-median, inverse-variance weighted (IVW), simple mode, and weighted mode were applied to assess the relationships and reported as odds ratio (OR) and 95% confidence interval (CI). The results of the IVW were used as the primary results, and the other MR methods were used as complements. Heterogeneity was measured by Cochran’s Q statistics, and a *P* value <0.05 for the Q statistics implied the presence of heterogeneity (18). Leave-one-out (LOO) analysis was applied to evaluate if the relationship was due to an individual SNP. In addition, the reverse causal relationship of exposure with outcome was assessed by bidirectional MR analysis.

To investigate pack-years of CS and alcoholic beverages/week on LC, multivariable MR analysis was performed (20). Multivariable MR is an extension of univariable MR to detect the causal effect of the combination of multiple risk factors (21). The IVW method was applied to multivariable MR analysis. A *P*-value of less than 0.05 was considered as significant statistically. R packages ‘TwoSampleMR’ and ‘MendelianRandomization’ were used for univariable MR analysis, and the ‘MendelianRandomization’ and ‘MVMR’ packages were used for multivariable MR analysis.

## Results

### Genetic instruments

A total of 14,853 SNPs were identified to related to 25(OH)D levels, with a significance level of *P* < 5 × 10^−8^. After screening, 118 SNPs did not contain LD, of which 107 SNPs were linked to the levels of 25(OH)D and LC. Following further screening, 92 SNPs with MAF > 0.01 were included in the analysis. Of these 92 SNPs, 89 SNPs were only linked to the levels of 25(OH)D, and the other SNPs were associated with the levels of 25(OH)D as well as with smoking or drinking. No horizontal pleiotropy was found for either of these 92 SNPs or 89 SNPs (*P* > 0.05) (Table 2). The *F*-statistics for these 92 SNPs and 89 SNPs were >10, suggesting the absence of weak instrument bias. In addition, the heterogeneity test showed that neither of these 92 SNPs nor the 89 SNPs were heterogeneous (*P* > 0.05).

**Table 2:** Testing of instrumental variables

SNP	Number of SNPs	Horizontal pleiotropy	Strength	Heterogeneity	
		MR-Egger (Intercept, *P*)	*F*-statistic, R^2^	MR-Egger (Q statistic, *P*)	IVW (Q statistic, *P*)
All SNPs	92	(0.0000, 0.2845)	32.974, 0.7	(81.7828, 0.7197)	(82.9418, 0.7143)
SNP0	89	(0.0000, 0.374)	37.203, 0.8	(74.8815, 0.8196)	(75.6800, 0.8225)

SNP: single-nucleotide polymorphisms; MR: Mendelian randomization; IVW: inverse-variance weighted; SNP0: SNPs associated with 25-hydroxyvitamin D levels only. The other SNPs, associated with 25-hydroxyvitamin D levels as well as smoking or drinking.

### Causality of 25(OH)D levels on LC risk

**Fig. 2:**
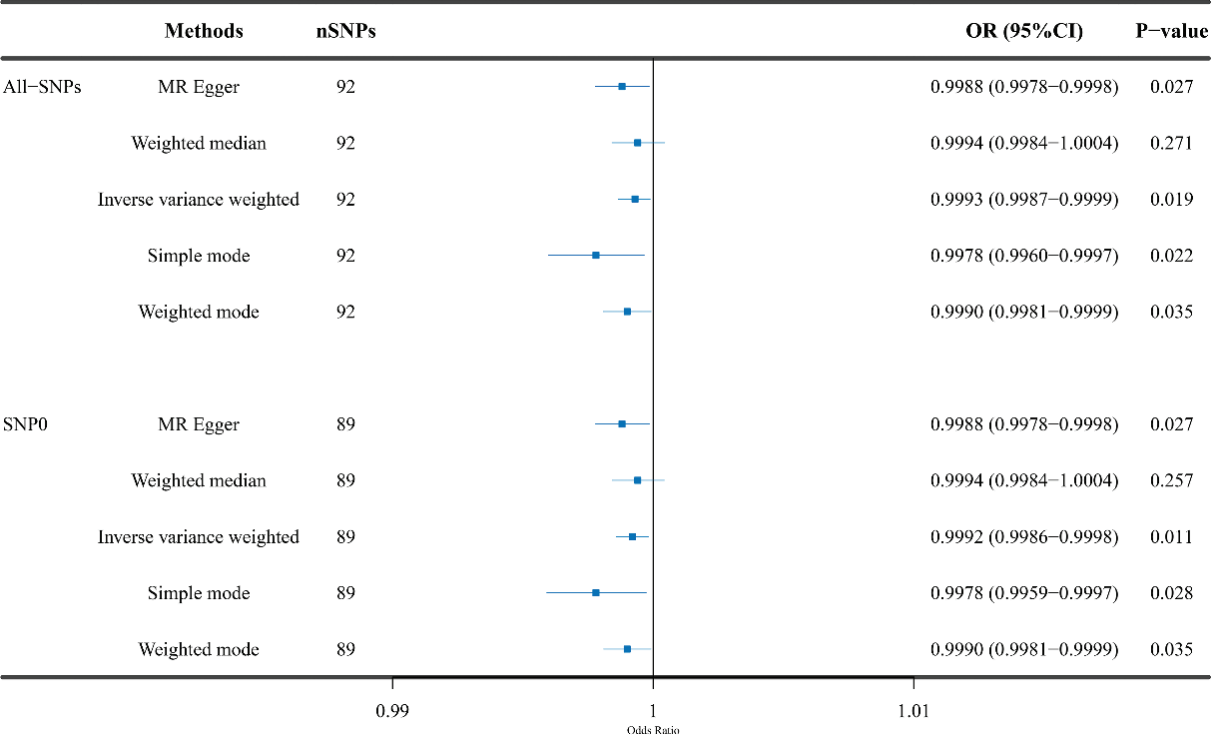
Univariable MR analysis of the causal association between 25-hydroxyvitamin D levels and laryngeal cancer risk. MR: Mendelian randomization; SNP0: SNPs associated with 25-hydroxyvitamin D levels only; the other SNPs, associated with 25-hydroxyvitamin D levels as well as smoking or drinking. SNP: single-nucleotide polymorphisms.

**Fig. 3:**
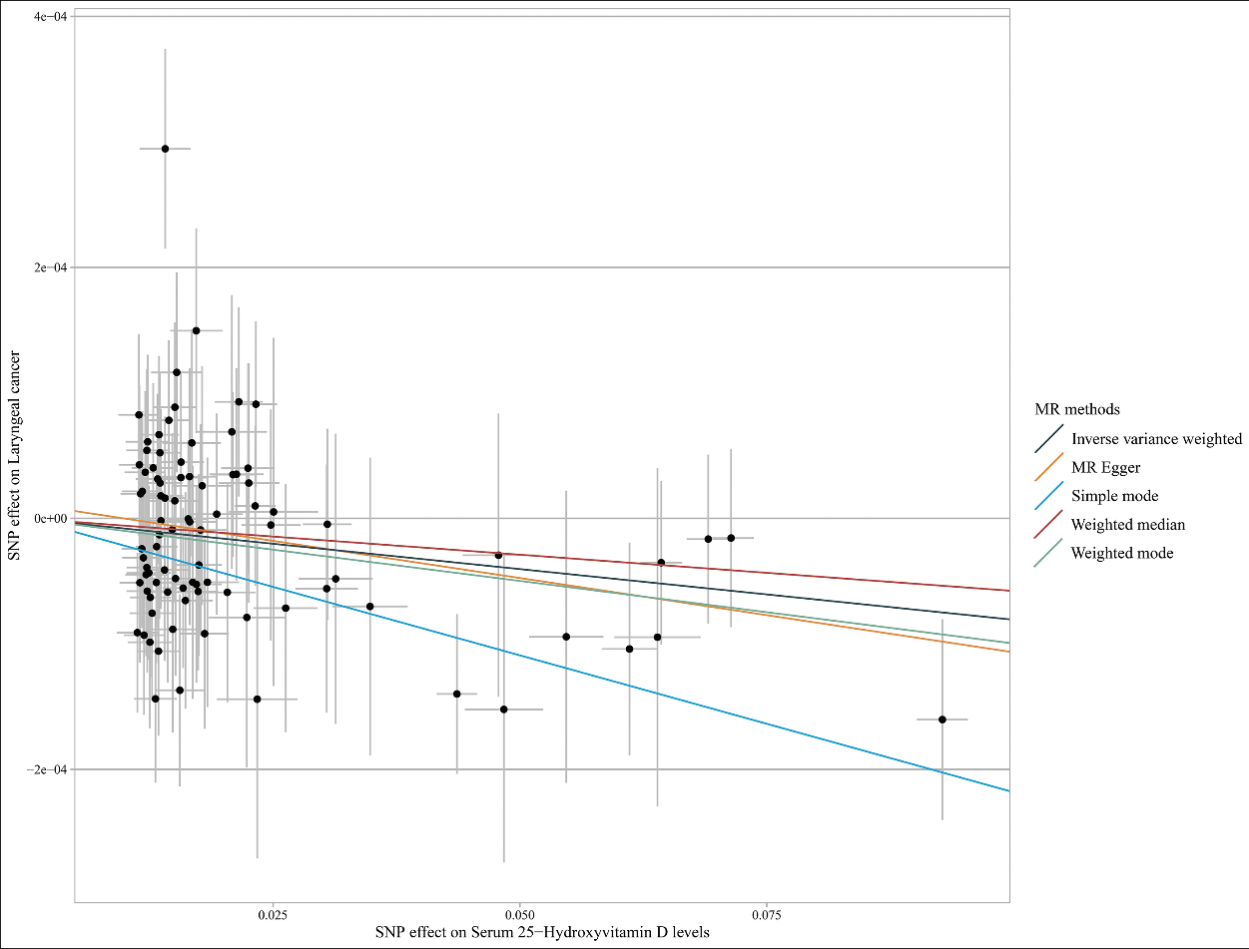
The scatter plots for the causal association between 25-hydroxyvitamin D levels and laryngeal cancer risk. SNP: single-nucleotide polymorphisms; MR: Mendelian randomization.

Figure 2 shows the univariable MR analysis of causality between the levels of 25(OH)D and LC risk. For these 92 SNPs, genetically predicted high levels of 25(OH)D decreased LC odds [IVW: (OR = 0.9993, 95% CI, 0.9987–0.9999; *P* = 0.019); MR-Egger: (OR = 0.9988, 95% CI, 0.9978–0.9998; *P* = 0.027); simple mode: (OR = 0.9978, 95% CI, 0.9960–0.9997; *P* = 0.022); and weighted mode: (OR = 0.9990, 95% CI, 0.9981–0.9999; *P* = 0.035)]. For these 89 SNPs, genetically predicted higher 25(OH)D levels were also correlated to a lower LC incidence [IVW: (OR = 0.9992, 95% CI, 0.9986–0.9998; *P* = 0.011); MR-Egger: (OR = 0.9988, 95% CI, 0.9978–0.9998; *P* = 0.027); simple mode: (OR = 0.9978, 95% CI, 0.9959–0.9997; *P* = 0.028); and weighted mode: (OR = 0.9990, 95% CI, 0.9981–0.9999; *P* = 0.035)]. The scatter plots for causality of 25(OH)D levels on LC were presented in Fig. 3.

**Fig. 4:**
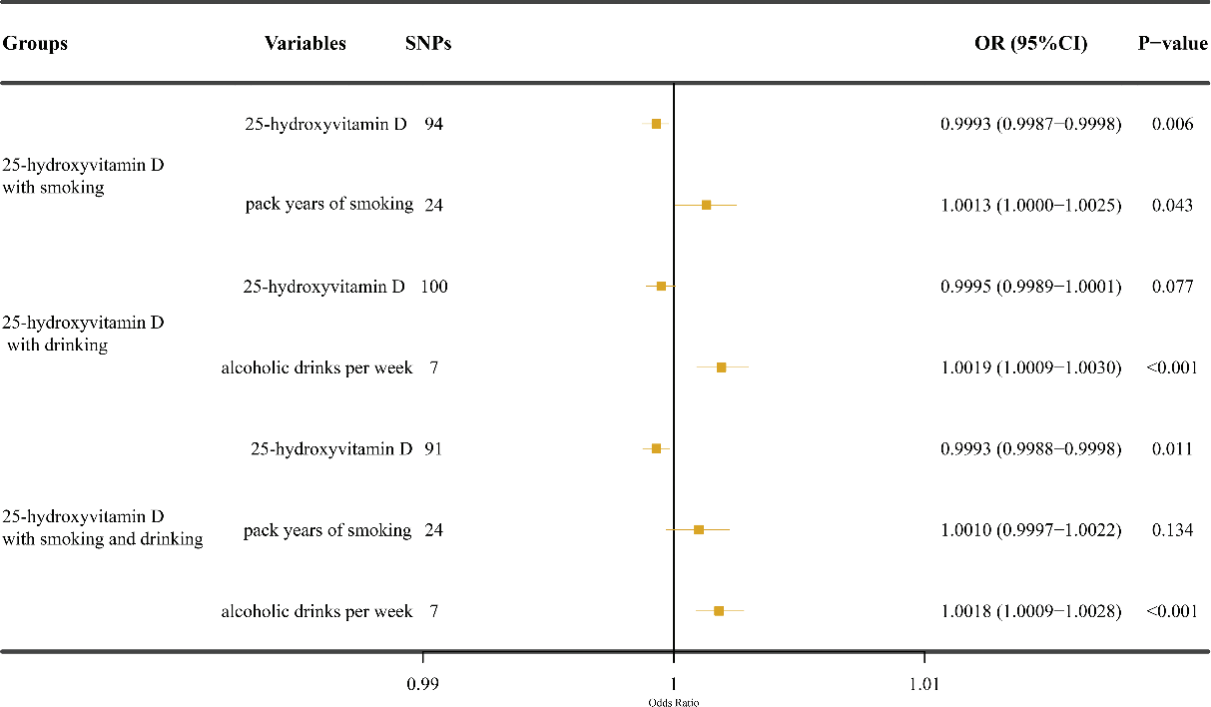
Multivariable MR analysis of the relationship between 25-hydroxyvitamin D levels and laryngeal cancer risk. SNP: single-nucleotide polymorphisms; MR: Mendelian randomization.

The multivariable MR analysis suggested that genetically predicted high 25(OH)D levels decreased LC risk after adjusting for pack-years of CS [IVW: (OR = 0.9993, 95% CI, 0.9987–0.9998; *P* = 0.006)] or both pack-years of CS and weekly alcoholic drinks [IVW: (OR = 0.9993, 95% CI, 0.9988–0.9998; *P* = 0.011)], while it was not substantial following adjustment for weekly alcoholic drinks [IVW: (OR = 0.9995, 95% CI, 0.9989–1.0001; *P* = 0.077)] (Fig. 4).

**Fig. 5:**
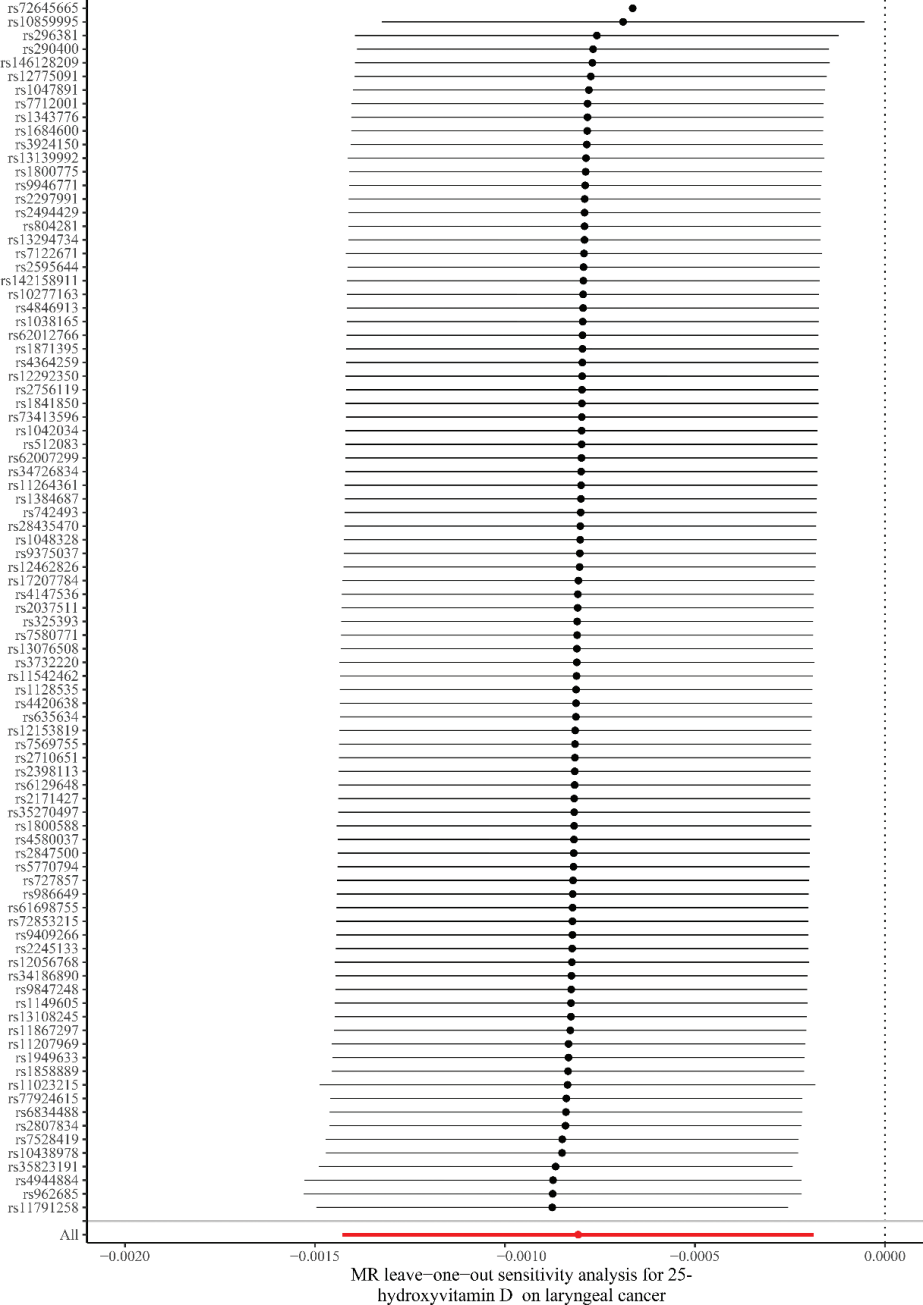
Leave-one-out plots for the relationship between 25-hydroxyvitamin D levels and laryngeal cancer. MR: Mendelian randomization.

LOO found that a causal link of 25(OH)D levels to LC could not be explained by any single SNP (Fig. 5). In addition, the bidirectional MR analysis reported no reverse causal link of 25(OH)D levels to LC risk (IVW: *P* = 0.345) (Table 3). The notably wide CIs reflect limited statistical power due to the small number of SNPs (*N* = 12) and low case counts of LC (*N* = 273).

**Table 3:** Bidirectional MR analysis between 25-hydroxyvitamin D levels and laryngeal cancer risk

MR methods	Number of SNPs	Beta (95% CI)	*P*
MR Egger	12	-	0.093
Weighted median	12	8.8656 (0.0484–1624.4057)	0.412
Inverse variance weighted	12	10.1728 (0.0828–1249.1583)	0.345
Simple mode	12	0.7701 (0.0001–8004.5739)	0.957
Weighted mode	12	0.8659 (0.0002–4207.4229)	0.974

SNP: single-nucleotide polymorphisms; MR: Mendelian randomization.

## Discussion

This study investigated the causal relationship of 25(OH)D levels to LC risk via MR analyses, finding that genetically predicted high levels of 25(OH)D decreased LC risk, even after adjusting for pack-years of CS or both pack-years of CS and weekly alcoholic drinks.

25(OH)D is a metabolite of VD and the main circulating form of VD in the body. Unlike variable environmental exposures (sunlight, diet, and supplements) or seasonally changing 25(OH)D levels (22), genetically predicted 25(OH)D levels are constant and do not change with disease (23). Various studies have reported a causal link of 25(OH)D levels to cancer risk (12, 23). A meta-analysis demonstrated that high levels of 25(OH)D were linked to an improved total survival and progression-free survival in cancer patients (23). Sutherland et al. reported an *L*-shaped relationship between genetically predicted 25(OH)D levels and mortality from cancer (24). However, a few MR studies showed that no causal link of 25(OH)D levels to the risk of cancer, including oral cavity (OC) and PC (12). MR analysis by Dudding et al. also supported the absence of a causal link between 25(OH)D levels and the risk of OC and PC (13). However, there is heterogeneity among different cancers. The current study used univariable MR and multivariable MR analyses to examine the causal link of 25(OH)D levels to LC risk. Our results supported a causal link of genetically predicted high levels of 25(OH)D to a decreased LC risk. Our study may provide new genetic insights into the association of 25(OH)D with LC risk. Potential mechanisms underlying the effects of 25(OH)D on cancer risk included: interference in autophagy, apoptosis, and differentiation of tumor cells, inhibiting proliferation, invasion, and metastasis, attenuating the proliferation and phenotypic characteristics of tumor stem cells, modulating various non-tumor stromal cells physiology (fibroblasts, endothelial cells), and activating immune cells and responses (25).

Sutherland et al. demonstrated that in cancer patients with 25(OH)D levels < 50 nmol/L, the death risk decreased dramatically with increasing 25(OH)D levels (24). This suggests a possible protection of dietary VD or VD supplements intake on LC risk and prognosis. In addition, alcohol and tobacco are major factors for LC risk, and tobacco and alcohol have a multiplicative effect on LC risk (26). In our multivariable MR analysis, we adjusted separately for pack-years of CS, weekly alcoholic drinks, and both pack-years of CS and weekly alcoholic drinks, finding that genetically predicted higher levels of 25(OH)D were correlated to a lower LC risk after adjusting for pack-years of CS or both pack-years of CS and weekly alcoholic drinks, but this was not significant after adjusting for weekly alcoholic drinks only. This result may be related to the amount of alcohol consumed, which we used weekly alcoholic drinks in analysis, and there is a linear relationship of alcohol consumption to LC risk (27).

Although our results supported a causal link of 25(OH)D levels to LC risk, the effect size is small. This indicates that genetically predicted 25(OH)D levels exert a slightly protective effect on LC. The clinical relevance of such a small effect size for an individual is limited. However, given the high prevalence of vitamin D (VD) insufficiency/deficiency globally, even a very modest reduction in relative risk at the population level could translate into a non-negligible number of potentially preventable LC cases. VD supplementation is generally safe, accessible, and inexpensive. Therefore, if confirmed by future research, optimizing population VD status could represent a low-risk, potentially cost-effective component of a broader strategy for cancer prevention, particularly for cancers like LC where modifiable risk factors (smoking, alcohol) are paramount. Notably, the protective association remained significant after adjusting for pack-years of smoking in the multivariable MR analysis. This suggests that the potential beneficial effect of higher 25(OH)D levels might operate, at least partly, independently of smoking exposure.

Translating this MR finding into clinical or public health practice requires substantial further investigation. Key next steps include: (1) Large-scale prospective cohort studies with serial measurements of 25(OH)D to confirm the observational association and assess dose-response relationships; (2) Well-designed RCTs testing whether VD supplementation reduces the incidence of LC or its precursors, particularly in high-risk populations (e.g. smokers, those with VD deficiency); (3) Mechanistic studies to elucidate the biological pathways by which VD might influence laryngeal carcinogenesis. Our study provides preliminary genetic evidence supporting a causal role, motivating these essential future investigations

While the bidirectional MR analysis showed no significant reverse causality (IVW *P* = 0.345), the extremely wide CIs (Table 3) preclude definitive conclusions. This imprecision stems from the inherent limitations of using LC as an exposure in MR: the limited number of available genetic instruments and the rarity of the outcome. Future studies with larger LC GWAS datasets are needed to validate this finding.

Several limitations should be considered. First, the study summary data of GWAS are from Europeans, and whether these results can extend to different populations requires further validation. Second, exposure-SNPs and outcome-SNPs should be derived from different samples, and the overlap of the samples between exposure and outcome in our study is unknown. Third, the unavailability of individual-level data makes it impossible to assess selection bias due to competitive risk factors.

## Conclusions

This study used a two-sample MR design with univariable/multivariable MR analyses to investigate the causal link of 25(OH)D levels to LC risk. Genetically predicted high 25(OH)D levels were slightly protective against LC risk, even after adjusting for pack-years of CS or both pack-years of CS and weekly alcoholic drinks. While the small effect size suggests limited individual clinical relevance, the high global prevalence of VD deficiency and the safety of supplementation warrant further investigation into its potential public health impact.

## Data Availability

The datasets generated and/or analyzed during the current study are available in the GWAS, https://gwas.mrcieu.ac.uk/.

## Declarations

Ethics approval and consent to participate: The present study was a secondary analysis based on data from previously published original studies, and the ethical approval and informed consent were obtained in the previously published original studies. The ethical approval and informed consent in this paper are not applicable.
